# Maschinelle Intelligenz – Evolution oder Lebensqualität

**DOI:** 10.1007/s00287-021-01382-8

**Published:** 2021-08-03

**Authors:** Hubert Österle

**Affiliations:** Universität St. Gallen, St. Gallen, Schweiz

## Abstract

Die maschinelle Intelligenz durchdringt und verändert alle Lebensbereiche. Die Integration der digitalen Services in Superapps verschiebt die Macht von Individuen, von konventionellen Unternehmen und von Staaten hin zu Internetgiganten, die ihre Ressourcen dafür einsetzen, die digitalen Dienste so weiterzuentwickeln, dass ihr Kapital und ihre Macht weiterwachsen. Auf diese Weise treibt das Kapital die soziotechnische Evolution.

Konsumerismus, psychische Erkrankungen, Wohlstandskrankheiten, politische Polarisierung, Machtverschiebung zu Konzernen u. a., negative Konsequenzen einer rein kapitalgetriebenen Entwicklung verlangen nach Steuerungsmechanismen im Sinne der Lebensqualität. Die riesigen Datensammlungen der digitalen Dienste ermöglichen es, die Treiber der Lebensqualität besser verstehen zu lernen, messbar zu machen und damit die soziotechnische Evolution zum Wohle der Menschen zu lenken. Darin liegen die Chancen einer Disziplin Life Engineering.

## Leben mit maschineller Intelligenz

Derzeit nutzen Menschen je nach Technikaffinität und Lebensumständen monatlich ungefähr 6 SmartPhone-Apps^1^ [1], in Einzelfällen aber bis zu 100. Das sind Apps wie z. B. Videoconferencing, Navigation und Payment. Stark wachsende Bereiche sind Gesundheit (Schlaf etc.), Wohnung (Beleuchtung usw.), Fahrzeug (Kollisionswarnung usw.), Verkehr (Fußgängererkennung etc.) und öffentliche Verwaltung (z. B. Steuererklärung).

In 10–20 Jahren werden Superapps (fast) alle Dienste zusammenfassen, die ein Individuum benötigt. Wir sind uns meist gar nicht bewusst, wie viele digitale Services uns in allen Lebensbereichen bereits heute, im Jahre 2021, begleiten. Sensorik, 5G, die Robotik, das maschinelle Lernen, die Integration (Standardisierung, Verknüpfung, Bereinigung) der Benutzerdaten und einfachere Mensch-Maschine-Kooperation werden bis zum Jahre 2030 leistungsfähige und umfassende Lebensassistenten aus einer Hand für Gesundheit, Mobilität, Arbeit, Unterhaltung usw. ermöglichen.

Je stärker die erwähnten Dienste zur Superapp einer dominanten Internetplattform zusammenwachsen, desto integrierter werden die Personen- und Sachdaten. Es entsteht ein so umfassendes, detailliertes, exaktes und aktuelles *Bild des Menschen* (Digital Twin, Quantified Self), wie es noch nie existiert hat. Es umfasst die Kontakte, die Termine, die Finanzen, die sportlichen Aktivitäten, das Schlafverhalten, die Interessen oder Umgebungsdaten wie die Luftqualität und enthält sogar Daten wie die Hautoberflächenspannung oder die DNA, für die der Mensch keine natürlichen Sinne besitzt.

Noch nie gab es ein derart breites und detailliertes Verhaltenswissen. Die Megaportale leiten aus den riesigen Datensammlungen *Verhaltenswissen* ab. Sie schlagen in der Navigation ungefragt die Route nach Hause oder ins Büro vor oder liefern uns aufgrund unseres Leseverhaltens oder unserer Freunde Nachrichten, die zu unserer politischen Ausrichtung passen.

Noch nie gab es eine derart enge *Kooperation zwischen Menschen und Maschinen*. Die Megaportale messen ihren Erfolg u. a. an der Zeit, die ein Benutzer mit ihren Services verbringt, also die Zeit, die zur Beeinflussung des Individuums zur Verfügung steht. Schon immer versuchten Politik, Religion und Unternehmen, das Verhalten der Menschen über Erziehung, Werbung, Vorschriften und Sanktionen zu lenken. Noch nie hatten sie allerdings ein derart ausgefeiltes Instrumentarium zur Verfügung. Menschen kommunizieren immer mehr mit ihren Maschinen als mit den Menschen, sodass die Maschinen das Verhalten der Menschen nach und nach steuern können, zu deren Vorteil wie zu deren Nachteil.

Die maschinelle Intelligenz verändert unser Leben so schnell und grundlegend, dass wir von einem *Evolutionssprung* sprechen müssen, keinem biologischen, aber einem *soziotechnischen*. Die digitalen Dienste assistieren den Menschen in allen Lebensbereichen und schaffen neue Formen der Produktion (z. B. globale Arbeitsteilung) und des Konsums von Gütern und Dienstleistungen (z. B. virtuelle Welten). Sie ersetzen oder verändern die Mechanismen der Gesellschaft (Meinungsbildung, Mitbestimmung, Bestrafung und Belohnung von Verhalten usw.).

Ein eher kleiner Teil der Menschen beschreibt vor allem die positiven Seiten der Technisierung, also beispielsweise das Besiegen von Krankheiten, die Beseitigung von Hunger, die Rettung der Umwelt und nicht zuletzt ein friedvolles Zusammenleben der Menschen [2, 3]. Weitaus mehr Menschen klagen primär über die Gefahren und haben Angst vor Überwachung, Stress durch Beschleunigung, Polarisierung und Hass, Komplexität und Überforderung, ja letztlich vor Entmenschlichung und Unterwerfung unter Maschinen. Doch auf welche digitalen Dienste wollen wir verzichten? Wie können wir dafür sorgen, dass die digitalen Dienste die Lebensqualität der Menschen erhöht?

Derzeit bestimmt eine viel zu emotionale Sicht auf die maschinelle Intelligenz die öffentliche und wissenschaftliche Diskussion. Wir benötigen eine Disziplin Life Engineering, welche die technologische Entwicklung erfasst, die Auswirkungen auf die Lebensqualität rational zu verstehen lernt und daraus Handlungsanleitungen für die Individuen, die Wirtschaft und die Gesellschaft ableitet. Dieser Aufsatz ist ein Versuch dazu.

*Die Entwicklung ist nicht aufzuhalten, sondern wird sich noch beschleunigen. Maschinelle Intelligenz, also die Menge aller digitalen Services, ermöglicht und schafft neue Mechanismen zur Steuerung von Individuen, Unternehmen und Gesellschaft. Eine Disziplin Life Engineering muss die Gefahren, vor allem aber die Chancen der maschinellen Intelligenz erkennen und sie zum Wohle der Menschen gestalten.*

## Der evolutionär gesteuerte Mensch

Wenn die Menschen die maschinelle Intelligenz zu ihrem Wohle nutzen wollen, müssen sie verstehen, was das Wohl (Glück, Freude) oder Wehe (Unglück, Leid), also die Lebensqualität, ausmacht. Damit beschäftigt sich die Philosophie schon seit Platon, zunehmend aber auch die Psychologie, Soziologie, Ökonomie, die Neurowissenschaften und die noch junge Glücksforschung. Zur Beurteilung von digitalen Services diene hier ein stark vereinfachtes Modell der Lebensqualität (zur Vertiefung siehe [4]), das den Menschen als lernendes System mit dem Ziel hoher Lebensqualität versteht.

**Figure 1:**
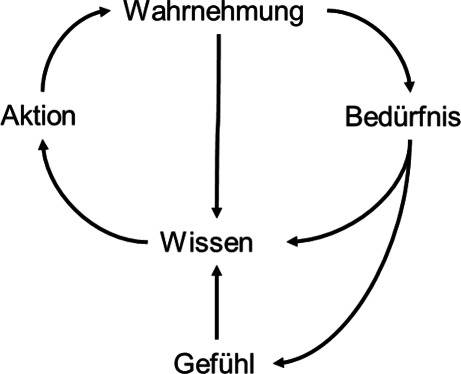

Jede Wahrnehmung trägt bewusst oder unbewusst zum Wissen des Menschen bei (siehe Abb. 1). Die Aktion „Post auf Instagram“ erzeugt beispielsweise die Wahrnehmung, wie oft der Post angeklickt wurde und wie viele Likes und Kommentare er erhält. Positive Rückmeldungen befriedigen das Bedürfnis nach Anerkennung (Status) und erzeugen ein Gefühl der Freude. Beleidigende Reaktionen bedienen dieselben Bedürfnisse, lösen aber Leid aus. Aktion, Wahrnehmung, Bedürfnis und Gefühl bilden einen Wissensbaustein (Verhaltensmuster) [5].

Die Bedürfnisse des Menschen sind teilweise ererbt und teilweise erworben. Das Bedürfnis nach Status innerhalb der Gemeinschaft ist ererbt und sorgt für die Selektion der besten Gene in der Fortpflanzung. Die Verbindung von Rückmeldungen in Instagram mit dem Streben nach Rang innerhalb der Gruppe (Status) wird durch die Sozialisation erworben und bildet ein Verhaltensmuster „Instagram Post“.

**Figure 2:**
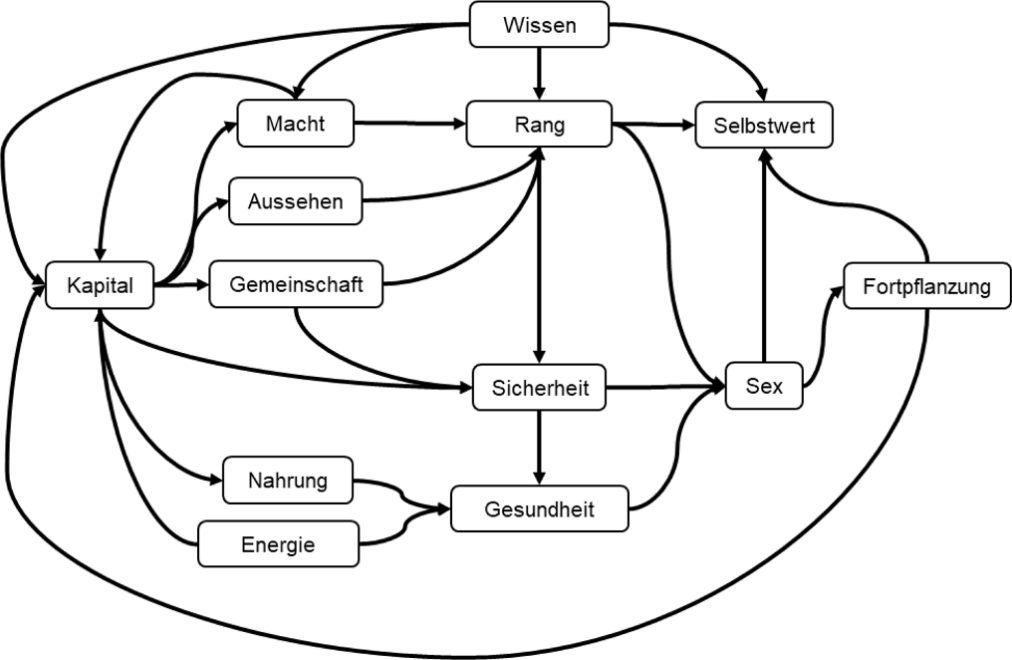

Abb. 2 fasst einige Erkenntnisse der Psychologie [6] und benachbarter Wissenschaften [7] zu 13 Bedürfnissen zusammen. Diese Auswahl von untereinander abhängigen Faktoren wirkt bei aller Sorgfalt, mit der sie zusammengetragen worden sind, willkürlich und grob, hilft aber, menschliches Verhalten zu erklären. Wenn die Grundbedürfnisse der Selbst- und Arterhaltung, beispielsweise Nahrung und Sicherheit, erfüllt sind, richtet der Mensch seine Kraft auf die Differenzierung von seinen Artgenossen etwa durch Aussehen, Macht oder Wissen. Er will seinen Rang erhöhen und so attraktivere Fortpflanzungspartner gewinnen. So sorgt die Evolution dafür, dass die stärksten Gene selektiert und weitergegeben werden. Das Glück der Menschen ist nicht das Ziel der Evolution, sondern das Mittel zur Steuerung des menschlichen Verhaltens.

Ein Problem der Bedürfnisbefriedigung liegt im Kampf zwischen Hedonia und Eudaimonia. Hedonia ist das unmittelbare und kurzfristige Gefühl, beispielsweise ein Erfolg in einem Videospiel. Häufig wird Hedonia mit Lust gleichgesetzt. Eudaimonia ist die längerfristige Zufriedenheit mit sich selbst und dem Lebenssinn (vor allem im Bedürfnis Selbstwert). Unbeschränktes Spielen von Videogames erzeugt kurzfristige Lust an kleinen Erfolgserlebnissen (vor allem Bedürfnis Macht), nimmt dem Spieler aber beispielsweise die Zeit zum Lernen oder zur Pflege von physischen Freundschaften und damit zur längerfristigen Zufriedenheit mit sich selbst.

Das Netzwerk der Bedürfnisse ist ein rudimentäres neuronales Netzwerk, das in viele Ebenen von neuronalen Netzen zu verfeinern ist. Datensammlungen helfen, Verhaltensmuster zu prüfen und weiterzuentwickeln. Die Megaportale leiten daraus das Verhaltenswissen, z. B. in Form des Knowledge Graphs von Google, ab, mit dem sie die Beeinflussung der Menschen in Werbung und Verkauf steuern. Das skizzierte Lebensqualitätsmodell mit den 13 Bedürfnissen kann als Aggregation unzähliger Verhaltensmuster gesehen werden. Die Komponenten und Zusammenhänge vollständig zu erkennen, ist eine Aufgabe, welche nicht nur den Menschen, sondern auch die heutigen Möglichkeiten des maschinellen Lernens bei Weitem überfordert. Wir müssen uns bis auf Weiteres mit eher einfachen und isolierten Verhaltensmustern wie etwa „Schlafqualität und Gesundheit“ zufriedengeben.

Damit kommen wir zu zentralen Fragen des Einsatzes der maschinellen Intelligenz zum Wohle der Menschen. Weiß das Individuum, was gut oder schlecht für seine Lebensqualität ist? Weiß die Wissenschaft, welches Verhalten zu Glück oder Unglück führt? Und wenn es der Mensch weiß, hat er die Konsequenz zur Umsetzung? Schaltet der Mensch beispielsweise für ein paar Stunden am Tag alle digitalen Kommunikationskanäle ab, wenn er weiß, dass er mehr Zeit für die Verarbeitung und weniger Zeit für die Aufnahme von Informationen aufwenden sollte, um zufriedener zu werden? Am Schluss stellt sich die Frage: Welche digitalen Dienste sollen entwickelt werden und wer realisiert in diesen seine Interessen?

*Die soziotechnische Evolution nutzt Glück und Unglück, um die Entwicklung zu treiben. Die Lebensqualität der Menschen ist nicht das Ziel, sondern das Mittel der Evolution. Die maschinelle Intelligenz erfasst immer detailliertere Daten jedes Menschen und leitet daraus ein Verhaltensmodell aus Bedürfnissen, Aktionen, Wahrnehmungen, Gefühlen und Wissen ab. Aus Sicht der Wirtschaft ist dies ein Konsummodell und aus Sicht der Ethik ein Lebensqualitätsmodell. Der Politik bietet das Verhaltensmodell neue Instrumente zur Steuerung der Gesellschaft.*

## Autonomie und Manipulation des Menschen

Wenn die Maschine besser als der Mensch weiß, welche Lebensmittel er kaufen sollte, kann sie ihm den Einkauf abnehmen. Wenn die Maschine aus den Persönlichkeitsmerkmalen den richtigen Partner fürs Leben findet, kann der Mensch die Auswahl der Maschine überlassen. Spätestens hier beginnt die Angst vor dem Verlust der Autonomie, der Punkt, an dem die Emotionen besonders hochgehen. Eine Befragung von 240 sogar weitgehend technikaffinen Personen hat dies deutlich gezeigt [8].

Die Angst vor dem Unbekannten und Unverstandenen ist stärker als die Vernunft. Autonomie (Freiheit, Souveränität usw.) ist eines der stärksten Anliegen der Ethik. Doch freier Wille ist eine seit eh und je heftig umstrittene Vorstellung, die gerade mit Blick auf die maschinelle Intelligenz erneut zu überdenken ist. Was treibt die Entscheidungen des Menschen? Es sind seine Bedürfnisse, die er durch seine Gene ererbt oder durch seine Umgebung und Erfahrung erlernt hat. Ererbte Bedürfnisse, die in unseren Genen angelegt sind, führen zu Gefühlen wie Liebe, Eifersucht, Rache und Lust. Wie das Verhalten der Menschen ständig zeigt, sind diese Gefühle meist stärker als die Vernunft, schränken also die freie Entscheidung des Menschen weitgehend ein.

Erlernte Bedürfnisse (Muster) entstehen zum Teil aus der persönlichen Erfahrung des Menschen. Wenn sich jemand in TikTok präsentiert, lernt er, dass sich seine Vergleichspersonen dort ebenfalls von der besten Seite zeigen. Die Entscheidung aufgrund der eigenen Erfahrungen kann man am ehesten als autonom bezeichnen, denn sie folgt den Verhaltensmustern, die der Mensch selbst erkannt hat. Letztlich sind es aber Muster, die aus den Genen und der Umgebung abgeleitet sind. Ein allgegenwärtiges erlerntes Bedürfnis ist das nach Kapital. Digitale Dienste bedienen dies beispielsweise mit Preisvergleichen und Verdienstmöglichkeiten etwa durch Posts.

Ein großer Teil der erlernten Bedürfnisse wird uns von unseren Mitmenschen vermittelt. Die Sozialisation in der Umgebung, in der wir aufwachsen und leben, vermittelt uns vielfach unbewusst Regeln und Werte, wie wir in der Gesellschaft erfolgreich leben. Die schulische Erziehung, die Religionen und die Ethik formalisieren die Verhaltensmuster, wie z. B. die Behandlung eines Geschäftspartners, und wollen unser Verhalten an ihre Vorstellungen anpassen. Die Medien, also Videos, Filme, Fernsehen, Zeitungen, Bücher usw. präsentieren uns Vorbilder (Influencer), von denen wir Werte (z. B. Statussymbole) und Verhaltensweisen übernehmen.

Die Konsumforschung hat ein vielfältiges Instrumentarium zur Manipulation hervorgebracht und die Digitalisierung liefert den Turbo dafür. Die omnipräsente Werbung kennt das generelle sowie das individuelle Kaufverhalten, unsere Präferenzen und unsere finanziellen Verhältnisse, sodass sie uns jene Produkte und Dienste anbietet, die den größten Verkaufserfolg erwarten lassen. Sie spricht dabei jene, uns selbst häufig unbewussten Bedürfnisse an, die uns zum Kauf verleiten. Wenn die Werbung unsere Finanzen, unseren Beruf und Freundeskreis, unsere Einkaufshistorie und unsere Foto- und Videosammlung kennt, kann sie beispielsweise einen prestigeträchtigen Urlaub auf den Bahamas in bunten Farben ausmalen.

Die sozialen Netze sind ein Ort der Selbstdarstellung und des meist unbewussten Vergleichs unter Freunden und Bekannten. Vermitteln die Einweg-Medien wie das Kino die Statussymbole von unerreichbaren Idolen, so stellen die sozialen Medien die Mitglieder einer Gemeinschaft nebeneinander, verstärken die Wirkung durch Interaktion (Kommentare, Applaus, Likes usw.) und treiben die Teilnehmer wie nie zuvor in einen Wettbewerb der Attraktivität (Bedürfnis Rang) durch eindrucksvolle sportliche Leistungen, durch den Erwerb von Statussymbolen (Bedürfnis Aussehen), durch Positionen in Politik und Vereinen sowie beruflichen Aufstieg (Bedürfnis Macht).

Die maschinelle Intelligenz forciert eine besondere Form der Beeinflussung, das Nudging. Dieses verwendet nicht starre Vorschriften und finanzielle Anreize, sondern gibt kleine, nichtmonetäre Anstöße (Nudges), um das Verhalten der Menschen zu beeinflussen. Das kann der Hinweis auf einen „guten Zweck“ sein, wenn beim Verkauf von Konzerttickets die Weitergabe von 10 % des Erlöses für die Ausbildung von Straßenkindern versprochen wird. Das kann aber auch das Ranking in einer Fitnessgruppe sein, die ihre Trainingsperformance ins Netz stellt. Nudging steht für die Ansprache aller nichtfinanziellen Bedürfnisse mit dem Ziel der Verhaltensbeeinflussung. Suyanto et al. [9] beschreiben Nudging als lifestyle-based social engineering und plädieren dafür als Ergänzung zu den staatlichen Zwangsmaßnahmen, beispielsweise bei der Umsetzung von Hygienevorschriften, wie sie mit Covid-19 vereinbart werden.

Das Social Scoring, das China in einigen Regionen erprobt, gibt ein gesellschaftlich erwünschtes Verhalten vor. Es ahndet säumiges Zahlungsverhalten oder den Verstoß gegen Verkehrsregeln und es belohnt die Tätigkeit in sozialen Einrichtungen und systemkonformes Verhalten. Das Social Scoring könnte gewissermaßen als Weiterentwicklung des Strafgesetzbuchs interpretiert werden. Es sanktioniert nicht nur Fehlverhalten, sondern belohnt auch erwünschtes Verhalten.

Das „Social Scoring“ der westlichen Demokratien verwendet das Zahlungsverhalten (z. B. Schufa), das Flensburger Zentralregister für Verkehrssünder, die Schadenshistorie des Fahrzeuglenkers bei der Versicherung, das Vorstrafenregister, Schul- und Arbeitszeugnisse, die Datensammlung des Employment Screening, die 360°Grad-Bewertung von Mitarbeitern oder die Informationen des Tenant-Screenings usw. Wie man aus den Erfolgsberichten der Polizei ableiten kann, überprüft diese in der Terrorabwehr auch systemgefährdendes Verhalten, indem sie die Surfhistorie im Internet, die Mitgliedschaft in bestimmten sozialen Netzwerken und die Benutzung des Dark Nets überwacht.

Die Wirkung der maschinellen Intelligenz zur Verhaltensbeeinflussung, insbesondere im Nudging, wird bisweilen als libertärer Paternalismus bezeichnet. Die Väter dieser Richtung, Thaler und Sunstein, wollen damit erreichen, dass der Mensch zu einem Verhalten gestupst wird, aber die Freiheit behält, sich gegen den Stupser zu entscheiden. Sie vertreten damit explizit das Ziel, dass nicht nur das Kapital, sondern alle Bedürfnisse zum Wohle des Menschen bedient werden. Sie gehen allerdings nicht darauf ein, dass das Verhaltenswissen, aufgrund dessen sich das Individuum allenfalls gegen den Nudger entscheidet, durch alle oben beschriebenen Beeinflussungen entstanden ist.

*Gene, Gesetze, Erziehung, Sozialisation, Medien, Werbung, Nudging und Social Scoring bestimmen die Bedürfnisse und den Handlungsspielraum des Menschen. Das digitale Abbild des Menschen verstärkt die Instrumente der Beeinflussung. Was bleibt von der Autonomie des Menschen? Wer bestimmt, wonach gesteuert wird?*

## Kapital als Steuerungsinstrument der Evolution

Wie ich die Grundgedanken zum Life Engineering vor 10 Jahren einer Gruppe von Wirtschaftsinformatikstudenten vorgestellt habe, reagierten diese mit Unverständnis, das ein Student wie folgt zusammenfasste: „Wenn die Menschen das konsumieren, was ihnen nützt, und wenn die Wirtschaft die digitalen Dienste anbietet, die von den Konsumenten gewünscht werden, dann ist für beide gesorgt und die Welt in Ordnung.“

Das erwartete Payback einer Investition entscheidet, welche Dienste und welche Unternehmen sich durchsetzen, der tatsächliche Payback liefert die Kraft für die Weiterentwicklung und Vermarktung. Das iPhone von Apple und die Suchmaschine Google von Alphabet sind die besten Beispiele dafür. Das Kapital wird zum Treibstoff der Evolution und hat die materielle Situation weiter Teile der Weltbevölkerung verbessert. Der Wettbewerb von Millionen von Entwicklern und Unternehmern zusammen mit den Entscheidungen von Milliarden von Konsumenten erzeugt eine Kreativität, die unseren materiellen Wohlstand überhaupt erst ermöglicht. Die libertären Denker betonen, dass die Menschen die Autonomie besitzen, für sich selbst zu entscheiden, was für sie am nützlichsten ist. Es gibt allerdings Gegenargumente:

- Die Digitalisierung erzeugt eine **Komplexität **[10], welche die Individuen überfordert, sodass sie die Konsequenzen vieler Entscheidungen nicht mehr abschätzen können. Das wird uns bei der Zustimmung zur Verwendung von Cookies und zu den Allgemeinen Geschäftsbedingungen eines digitalen Service immer wieder vor Augen geführt. Welcher Konsument versteht denn noch die Deckungen seiner Fahrzeugversicherung, die die Versicherer aus der Analyse riesiger Schadensdatenbanken und allenfalls von Persönlichkeitsmerkmalen abgeleitet haben, um ihr Schadensrisiko zu minimieren?
- Unternehmen und Konsumenten haben teilweise **konkurrierende Ziele**. Der RoboAdvisor einer Bank empfiehlt ein derivatives Produkt der Bank, welches das Bedürfnis des Kunden nach einem schnellen Zuwachs des Kapitals (Gier) anspricht. Der Kuchen, der zwischen Bank und Kunde zu verteilen ist, bleibt aber immer gleich. Wenn dann noch eine Erfolgsbeteiligung der Bank ins Spiel kommt, die nur bei Gewinnen und nicht bei Verlusten schlagend wird, geht die Bank u. U. Risiken ein, die einmal zu hohen Gewinnen für die Bank und den Kunden, ein andermal aber zu hohen Verlusten nur für den Kunden führen.
- Das Kapital erzeugt fast zwangsweise **Konsumerismus** mit allen Konsequenzen. Die Digitalisierung fördert den Vergleich mit Freunden und Vorbildern und stellt die Menschen damit in der Freizeit wie im Beruf in das Hamsterrad der Evolution durch Selektion. Aus Sicht des Anbieters bedeutet mehr meist auch besser. Begleiterscheinungen sind Umweltzerstörung, Stress, Verschuldung sowie gesundheitliche Schäden wie Adipositas und Depression bis hin zum Drogenkonsum. Hedonia durch Chats in sozialen Medien ist stärker als Eudaimonia durch Meditation.
- Die Digitalisierung verändert die **Lebensgewohnheiten**. Ständige Erreichbarkeit, Verlust der Trennung von Beruf und Privatleben, E‑Mail und Social Networking bis spät in die Nacht, aber auch Unterhaltung durch Videos und Gaming, verändern den Lebensrhythmus. Das schlägt sich nicht selten in Schlafproblemen und als Folge davon in depressiven Stimmungen nieder.
- Die digitalen Medien prägen immer stärker das Weltbild und die **Werte** der Konsumenten. Sie vermitteln uns nicht nur Statussymbole, die unseren Konsum anregen, sondern auch Inhalte (Meinungen), die zu unserer Einstellung passen und diese damit verstärken. Sie tragen mit Filterblasen zur Polarisierung bei, wie beispielsweise der amerikanische Wahlkampf 2020 gezeigt hat. Um derartigen Gefahren entgegenzuwirken, schlägt u. a. Stray vor, die Algorithmen der maschinellen Intelligenz auf die Bedürfnisse von Gemeinschaften auszurichten [11]
- Digitale Services, ob sie die Suche von wissenschaftlichen Publikationen, die maschinelle Übersetzung oder die Navigation betreffen, nehmen dem Menschen Kompetenz und Entscheidungen ab, sodass er von den Services **abhängig** wird.
- Ein noch viel zu wenig beachtetes und verstandenes Phänomen ist die **Machtverschiebung **[12]. Technologiegiganten wie Facebook, Apple, Microsoft, Amazon, Netflix und Google, aber auch Baidu, Ant, JD, Alibaba und Tencent setzen sich in vielen Belangen gegen sogenannte souveräne Staaten durch, gleich ob es um Steuern, Datenschutz oder Verbraucherschutz geht. Sie besitzen jeweils fast monopolartige Macht und verhindern in ihren Ecosystemen das Aufkommen von kleinen, innovativen Unternehmen [13]. Sogar ein global tätiger Konsumgüterkonzern muss sich damit beschäftigen, ob Amazon seine Produkte durch Eigenmarken ersetzen kann und er damit u. U. nur noch zum Lieferanten von niedrigmargigen Massenwaren wird. Ein Autohändler berichtet davon, dass seine Gebrauchtwagen in Google praktisch nicht auffindbar sind, wenn er nicht bereit ist, für jeden Klick auf seine Website einen Betrag an Google zu überweisen. Mit ihren Mitteln zur Meinungsbildung bzw. -beeinflussung gefährden die Megaportale die Demokratie und den Markt.

*Die soziotechnische Evolution treibt über das Kapital die Entwicklung von Wissen, Technik und Regeln für Wirtschaft und Gesellschaft. Der wirtschaftliche Erfolg eines digitalen Service entscheidet, ob er entwickelt und betrieben wird. Wenige Megaportale bestimmen die Entwicklungsrichtung.*

## Lebensqualität als Steuerungsinstrument des Menschen

Utilitaristen wie Jeremy Bentham gehen davon aus, dass Glück das oberste Ziel der Menschen ist. Die Menschen versuchen, all ihre Bedürfnisse möglichst intensiv und dauerhaft zu befriedigen. Dies ist auch die Botschaft von Thaler und Sunstein, die durch Nudging die Aufmerksamkeit der Menschen auf alle Bedürfnisse lenken wollen. Die Reduktion auf einen Teil der Bedürfnisse wie Kapital, Macht und Gesundheit erzeugt Defizite bei den vernachlässigten Bedürfnissen. Da der Mensch ein ausgeprägtes Bedürfnis nach Arterhaltung (Gesundheit, Sex, Fortpflanzung, Sicherheit, Gemeinschaft) besitzt, liegt ihm das Gemeinwohl oder mindestens das Wohl seiner Gemeinschaft am Herzen. Die Ausrichtung an den Bedürfnissen bedeutet daher ganz im Sinne des Utilitarismus nicht reinen Egoismus, sondern Lebensqualität für alle, mindestens aber die eigene Gemeinschaft.

Diese Interpretation der Lebensqualität entspricht der Vorstellung, dass die soziotechnische Evolution nicht nur die stärksten Exemplare der Menschen selektiert, sondern dass sich jene Technologien und Organisationsformen durchsetzen, die der Menschheit als Ganzes den größten Nutzen bringen. Nach diesem Prinzip sind auch die komplexen Organisationen von Termitenbauten oder Bienenvölkern entstanden, da die Evolution nicht allein das Individuum, sondern die Gattung entwickelt.

Sämtliche ethischen Prinzipien lassen sich auf die hier behandelten 13 Bedürfnisse abbilden, wenn wir zur notwendigen Abstraktion bereit sind. Ungleichheit kann beispielsweise bedeuten, dass ein Individuum durch das Kapital oder die Macht anderer gehindert wird, ein bestimmtes Krankenhaus aufzusuchen, dass deswegen die Bedürfnisse Rang und Selbstwert leiden und u. U. das Bedürfnis Gesundheit nicht befriedigt werden kann. Das ethische Prinzip der Gleichheit schützt diese Bedürfnisse, widerspricht aber den Bedürfnissen der Menschen, die von einer Besserstellung profitieren.

Ein stark verfeinertes Modell der Lebensqualität könnte es erlauben, die vielerorts diskutierten Prinzipien der Digitalethik (siehe Beitrag *Spiekermann* in diesem Heft) zu operationalisieren, zu konkretisieren und letztlich durch konkrete Handlungsanweisungen zu ersetzen. Ein elaboriertes Modell der Lebensqualität kann aber auch die Ansätze der positiven Psychologie, die Erkenntnisse der Neurowissenschaften, ja sogar bewährte Konzepte von Religionen wie die zehn Gebote oder die buddhistische Meditation zu einer Anleitung zur Eudaimonia, also zum dauerhaften Glück durch Zufriedenheit mit sich und der Umwelt zusammenfassen und die Basis für einen digitalen Lebensassistenten bilden, der uns beobachtend und intervenierend durch das tägliche Leben begleitet.

*Soll die maschinelle Intelligenz die Lebensqualität erhöhen, so muss sie aus den Verhaltensdaten ein operationalisierbares Modell der Lebensqualität „lernen“ und anhand dessen die Individuen, die Unternehmen und die Gesellschaft anleiten.*

## Life Engineering

**Figure 3:**
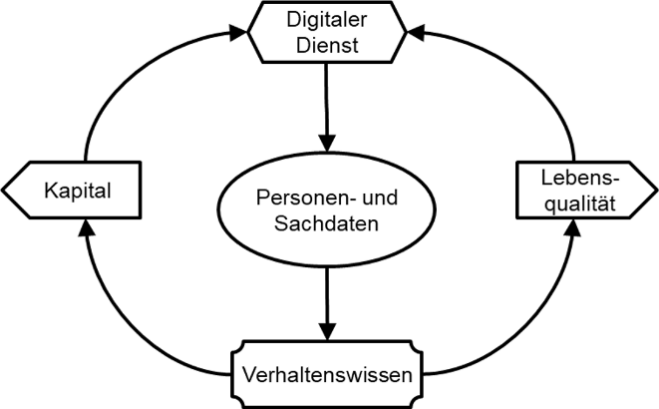

Die Menschheit steht vor der Wahl, die soziotechnische Evolution am Kapital oder aber an der Lebensqualität auszurichten (Abb. 3).

Das Kapital ist bis heute der wirkungsvollste Treiber der technologischen Entwicklung. Die Megaportale nutzen ihre Datensammlungen, ihre Modelle des Kaufverhaltens, ihren dominanten Kundenzugang und ihr Kapital, um ihre Dienste weiterzuentwickeln. So bieten sie ihren Kunden einen überlegenen Nutzen und steigern damit den Unternehmenswert, wie dies von ihren Aktionären gefordert wird. Was hilft es, wenn ein Unternehmen die Lebensqualität in den Mittelpunkt stellt, damit aber den Antrieb und die Ressourcen für die Entwicklung verliert, sodass kapitalgetriebene Unternehmen eine überlegene Technologie entwickeln, die früher oder später von allen übernommen werden muss (siehe dazu auch den Beitrag von *Falk* und *Riemensperger* in diesem Heft). In der technologischen Entwicklung gilt das Prinzip: Führe oder werde geführt.

In den letzten Jahren verfolgen zahlreiche Initiativen die Lebensqualität der technologischen und ökonomischen Entwicklung. Beispiele sind das Ethically Aligned Design mit den IEEE 7000™ Standards, die ISO Norm 26000 zur Corporate Social Responsibility, die ESG-Kriterien (ecological, social, governance) der OECD [14], Datenschutz, Verbraucherschutz und in letzter Zeit die europäische oder nationale Gesetzgebung zur Digitalisierung wie die DSGVO (Datenschutz-Grundverordnung). Sie zielen alle darauf ab, die eindimensionale Steuerung der soziotechnischen Evolution durch das Kapital um weitere Dimensionen der Lebensqualität zu erweitern.

Die Initiativen leiden daran, dass keine belastbaren Modelle der Lebensqualität vorhanden sind, dass die Ethik wenig operationalisierbare und damit überprüfbare Werte liefert, dass der Marktzugang für innovative Dienste mit der Lebensqualität im Zentrum meist aussichtslos ist und dass die Marktdominatoren ihre Interessen vielfach selbst in der Politik durchzusetzen vermögen.

Die Digitalisierung ist nicht nur eine gewaltige Herausforderung, sondern bietet ein wirkungsvolles Instrumentarium, die kapitalgetriebene Steuerung von Wirtschaft und Gesellschaft um viele Aspekte der Lebensqualität zu erweitern. Eine Disziplin Life Engineering kann dazu die Grundlagen liefern.

### Datenzugriff

Anbieter digitaler Dienste mit dem Ziel der Lebensqualität müssen den gleichen Zugang zu den Daten bekommen, wie ihn heute Dienste mit einem ausschließlichen Kapitalziel besitzen. Der Artikel 20 der DSGVO verlangt, dass ein Dienstanbieter auf Verlangen des Benutzers dessen Daten an einen anderen Dienstanbieter weitergeben muss. Im Idealfall könnte ein neuer Dienst Zugang zu allen Daten aller digitalen Dienste eines Nutzers bekommen. Eine Studie des BIDT (siehe Beitrag von *Kuebler-Wachendorff et al.* in diesem Heft) berichtet dagegen, dass das Recht auf Datenportabilität in praxi weitgehend wirkungslos ist, da die exportierenden Dienstanbieter die Datenweitergabe erschweren, die importierenden Dienstanbieter kaum automatisierte Datenübernahme anbieten und die Benutzer wenig über ihr Recht wissen. Das Privacy Paradox [15], nach dem Benutzer persönliche Daten beispielsweise in sozialen Netzwerken fast bedenkenlos preisgeben, gleichzeitig aber die Wichtigkeit des Datenschutzes betonen, erschwert den Neueintretern im Markt den Datenzugriff.

Die DSGVO differenziert nicht zwischen historischen Daten, die in den Datenbanken der Dienste liegen, und den Echtzeitdaten, die zum Zeitpunkt der Nutzung eines Dienstes entstehen. Ein digitaler Dienst kann zwar auf die API des Apple HealthKit oder des Google Fit zugreifen, ist dann aber auf die von Apple oder Google bereitgestellten Daten (Herzrhythmus, Bewegung usw.) beschränkt. Will eine neuartige App beispielsweise den Zusammenhang zwischen Onlineverhalten des Nutzers und seinem Wohlbefinden herstellen, braucht sie neben Daten wie dem Herzrhythmus unter anderem Informationen über die genutzten Funktionen (z. B. bei der Organisation einer Bahnreise) oder das Bedienverhalten (z. B. Fehler bei der Eingabe).

Dienstanbieter kämpfen zusätzlich mit der Schwierigkeit inkompatibler Datenwelten. Ist es schon mühsam, syntaktisch unterschiedlich strukturierte Daten zusammenzuführen, ist es noch viel anspruchsvoller, die Daten anderer Dienste semantisch richtig zu interpretieren, selbst wenn sie mit aufwändigen Ontologien dokumentiert sind. Deshalb will die Europäische Union mit GAIA‑X, basierend auf der IDS-Software-Infrastruktur, Datenräume für konkrete Ökosysteme schaffen (siehe Beitrag von *Otto und Burmann* in diesem Heft). Für die europäische Wirtschaft wie für die Verbraucher von besonderer Bedeutung ist der Mobilitätsdatenraum, der das Ökosystem Mobilität auf ein neues Niveau heben soll.

### Verhaltenswissen

Digitalisierung zum Wohle der Menschen setzt voraus, dass wir wissen, was dieses Wohl ausmacht. Die riesigen Datensammlungen der digitalen Dienste versetzen uns in die Lage, Verhaltensmuster und damit verbundene Indikatoren für die Lebensqualität zu untersuchen. Die Entwicklung eines operationalisierbaren Lebensqualitätsmodells kann auf viele Erkenntnisse, beispielsweise der Psychologie [16, 17], zurückgreifen, eine systematische Suche nach Mustern, die alle oben angesprochenen Faktoren der Lebensqualität einbezieht, könnte sich zwar als bahnbrechende Opportunität der Digitalisierung herausstellen, steckt aber bislang bestenfalls in den Kinderschuhen.

Dagegen unterhalten die Megaportale riesige KI-Abteilungen, die neben dem Fachwissen der involvierten Wissenschaftsbereiche alle Techniken des maschinellen Lernens nutzen, um Verhaltensmuster im Konsum abzuleiten. Sie betrachten dieses Wissen als Kern ihres Geschäftsmodells und setzen es daher im Wettbewerb ein.

### Digitale Dienste

Internetnutzer verwenden gewöhnlich nicht mehr als 5–10 Apps zusätzlich zu den in iOS und Android vorinstallierten [18]. Sollen digitale Dienste mit dem Ziel der Lebensqualität die Benutzer erreichen, müssen sie Zugang zum Kunden erhalten, was auf den Widerstand der Platzhirsche stößt, wie beispielsweise der Rechtsstreit zwischen dem Spielehersteller Epic Games und Apple sowie Google [19] zeigt.

### Politische Implementierung

Der skizzierte soziotechnische Evolutionssprung verändert unsere Gesellschaft derart grundlegend, dass wir alle gesellschaftlichen Kräfte benötigen, um die wissenschaftlichen Erkenntnisse einer Disziplin Life Engineering wirksam zu machen, wenn wir die Entwicklung zum Wohle der Menschen und nicht nur des Kapitals steuern wollen. Es geht um Meinungsbildung, um Ausbildung, um politische Programme von Parteien, um Konsumentenschutzgesetzgebung, um zivilgesellschaftliche Aktivitäten und um internationale Abstimmung, da einzelne Staaten nicht gegen die Macht der Megaportale ankommen.

*Life Engineering liefert die wissenschaftliche Grundlage für gesellschaftliches Handeln.*

## Datennutz statt Datenschutz

Die DSGVO wird teilweise als europäische Vorzeigeleistung gefeiert und teilweise als bürokratisches Monster zum Vorteil der Megaportale anstelle der Bürger [20] verdammt. Möglicherweise sollte der Datenschutz grundsätzlich durch Datennutz ersetzt werden. Nicht die Sammlung von Daten ist zu begrenzen, sondern die Nutzung. Ein Modell könnte sein, dass Datentreuhänder den Zugriff auf sämtliche Personendaten für alle Anbieter von digitalen Services mit Zustimmung der betroffenen Person ermöglichen, dass dann aber kontrolliert wird, ob die Daten nur in dem Sinn verwendet werden, den der Dienstanbieter nach staatlichen Vorgaben deklariert hat. Wirtschaft und Wissenschaft arbeiten seit Längerem an derartigen Konzepten („self-sovereign identity“) und Lösungen [21]. Welchen Wert Personendaten haben, lässt sich daraus erahnen, dass Google jährlich 8–12 Mrd. USD an Apple dafür bezahlt, dass Apple in iOS die Google-Suchmaschine vorinstalliert [22].

Wie dringlich weniger Emotion und mehr Rationalität in der Diskussion sind, zeigt das aktuelle Beispiel der eID in der Schweiz. Das Schweizer Parlament hat beschlossen, eine EU-kompatible staatlich überwachte Identifikation zu schaffen, den Betrieb des Identifikationsservices aber privaten Anbietern zu überlassen. Gegen diese privatwirtschaftliche Lösung wurde das Referendum ergriffen, das im März 2021 vom Volk angenommen wurde, sodass der Datennutz aus der eID in der Schweiz wahrscheinlich um Jahre verzögert wird. Und dies in einem Land mit 8,6 Mio. Einwohnern, von denen 96 % im Internet aktiv sind, 6,5 Mio. einen E‑Mail-Account bei Google, Apple usw. besitzen, sich 4,5 Mio. in sozialen Netzwerken wie Facebook engagieren [23] und so ein Vielfaches der Daten ohne staatliche Kontrolle an kapitalgetriebene Unternehmen und außerhalb des Schweizer Hoheitsgebiets preisgeben.

Bedeutet Life Engineering mehr staatliche Regulierung? Ohne gesellschaftlichen Konsens wird es nicht gehen, aber ohne marktwirtschaftliche Mechanismen auch nicht. Es gilt, das Kapital als Treiber der Entwicklung zu nutzen und gleichzeitig die Ziele der Lebensqualität zu verfolgen.

## Nachsatz

Eine Disziplin Life Engineering stellt uns vor enorme emotionale Herausforderungen. Wir müssen akzeptieren lernen, dass umfassenden Datensammlungen und Maschinenlernen fundierte Erkenntnisse zum Verhalten der Menschen liefern können, auch wenn diese zunächst nur sehr einfache Muster, wie beispielsweise zu Videogaming und Schlafqualität, betreffen. Wir müssen akzeptieren lernen, dass viele Einflüsse unsere scheinbar autonomen Entscheidungen bestimmen, dass derzeit die Internetgiganten die größten Datensammlungen, das meiste Verhaltenswissen und die Kanäle zur Monetarisierung dieses Wissens besitzen und uns damit im Sinne des Kapitals steuern. Wir müssen die Chance erkennen, die wir als Gesellschaft haben, dieselben Ressourcen zum Wohle der Menschen einzusetzen.

Wenn Sie, lieber Leser, den Überlegungen zum Life Engineering wenigstens in Teilen zustimmen, dann gestatten Sie mir 2 letzte Fragen: Wer hat das Recht zur Steuerung der Menschen? Und wer hat die Pflicht zur Steuerung der Menschen? Ein empirisches Stimmungsbild zu diesen und ähnlichen Fragen findet sich auf www.lifeengineering.ch.

## Footnotes

1 neben den durch das Betriebssystem vorinstallierten.
